# A Computational Approach for Identifying the Chemical Factors Involved in the Glycosaminoglycans-Mediated Acceleration of Amyloid Fibril Formation

**DOI:** 10.1371/journal.pone.0011363

**Published:** 2010-06-29

**Authors:** Elodie Monsellier, Matteo Ramazzotti, Niccolò Taddei, Fabrizio Chiti

**Affiliations:** 1 Dipartimento di Scienze Biochimiche, Università di Firenze, Firenze, Italy; 2 Consorzio interuniversitario “Istituto Nazionale Biostrutture e Biosistemi” (I.N.B.B.), Roma, Italy; University of North Dakota, United States of America

## Abstract

**Background:**

Amyloid fibril formation is the hallmark of many human diseases, including Alzheimer's disease, type II diabetes and amyloidosis. Amyloid fibrils deposit in the extracellular space and generally co-localize with the glycosaminoglycans (GAGs) of the basement membrane. GAGs have been shown to accelerate the formation of amyloid fibrils *in vitro* for a number of protein systems. The high number of data accumulated so far has created the grounds for the construction of a database on the effects of a number of GAGs on different proteins.

**Methodology/Principal Findings:**

In this study, we have constructed such a database and have used a computational approach that uses a combination of single parameter and multivariate analyses to identify the main chemical factors that determine the GAG-induced acceleration of amyloid formation. We show that the GAG accelerating effect is mainly governed by three parameters that account for three-fourths of the observed experimental variability: the GAG sulfation state, the solute molarity, and the ratio of protein and GAG molar concentrations. We then combined these three parameters into a single equation that predicts, with reasonable accuracy, the acceleration provided by a given GAG in a given condition.

**Conclusions/Significance:**

In addition to shedding light on the chemical determinants of the protein∶GAG interaction and to providing a novel mathematical predictive tool, our findings highlight the possibility that GAGs may not have such an accelerating effect on protein aggregation under the conditions existing in the basement membrane, given the values of salt molarity and protein∶GAG molar ratio existing under such conditions.

## Introduction

Aggregation of proteins in the form of extracellular amyloid fibrils is a consistent mechanism underlying a group of diverse human diseases, including neurodegenerative disorders and non-neuropathic conditions [1]. These disorders differ for the type of protein undergoing aggregation, for the type of organs involved in amyloid deposition and, consequently, for the clinical profile featured in each case. Among the most prominent neurodegenerative conditions are Alzheimer's and Creutzfeldt-Jakob diseases, which affect the central nervous system via extracellular deposits of the amyloid β peptide and prion protein, respectively [1]. Examples of non-neuropathic conditions are light chain amyloidosis and hemodialysis-related amyloidosis, where deposits are found in joints, skeletal tissue, heart, kidney, etc. In these two cases the proteins involved are the immunoglobulin light chain and β2-microglobulin, respectively [1].

Amyloid fibrils are often localized in close proximity to basement membranes, a specialized component of the extracellular matrix that is mainly built of collagen and glycosaminoglycans (GAGs) [2]–[4]. GAGs are long unbranched polysaccharides that often occur as O- or N- linked side chains of proteoglycans, with the exception of hyaluronic acid existing in a free form. Naturally occurring GAGs include heparin, heparan sulfate, dermatan sulfate, keratan sulfate, chondroitin sulfate and hyaluronic acid. Other non-physiological derivatives of natural GAGs have been used for studies *in vitro*, such as fully-O-desulfated heparin and dextran sulfate [5]–[6]. GAGs have been found intimately associated with all types of amyloid deposits *in vivo* so far analyzed [7]–[14], leading to the hypothesis that they have fundamental relevance in amyloidogenesis [2], [4], [15]. More importantly, GAGs have been attributed an active role in amyloidogenesis, as they display an ability to promote fibrillogenesis *in vitro* for a number of protein or peptide systems [5], [6], [16]–[26]. The proteoglycan perlecan, in particular, has been implicated as an important factor determining amyloid fibril formation [2]–[4]. The active role of GAGs and proteoglycans in amyloid fibril formation in vivo has also been supported by the observation that inhibitors of heparan sulfate proteoglycan synthesis can reduce amyloid formation [27], [28].

Studies on the effect of GAGs on amyloid fibril formation have consisted so far on investigations focusing on a single protein, and on one or a limited number of GAGs. This has allowed the effect of one or more GAGs to be studied only on one particular system and in well defined experimental conditions. Nevertheless, the generic ability of GAGs to influence the process of amyloid fibril formation, independently of the GAG used, protein studied and solution conditions employed, encourages a systematic study using a heterogeneous database reporting different GAGs and protein systems and a variety of solution conditions. In this study we have collected all the experimental data so far published on the effect of GAGs on amyloid fibril formation *in vitro*. The data include different GAGs, proteins and experimental conditions and have been reported by different investigators. Using a number of single parameter studies, as well as a multivariate analysis, we have studied the database as a whole. We have identified the generic chemical determinants responsible for the GAG-mediated acceleration of amyloid fibril formation, and have used this knowledge to build a predictive equation of the effect of GAGs on protein aggregation.

## Methods

### Data collection

Articles were collected from PubMed using the keywords “(protein OR peptide) AND (aggregation OR amyloid OR fibrillation) AND (GAG OR glycosaminoglycan OR proteoglycan OR heparin OR heparan)”. Among the articles retrieved, only those presenting both kinetic data of aggregation *in vitro* and a clear explanation of the experimental conditions used to obtain such data were kept for further analysis. Experimental conditions include the nature and molar concentrations of the protein and GAG, and the precise characteristics of the milieu (composition, pH and temperature). Experiments performed in the presence of additional parameters susceptible to have important effects on the aggregation kinetics in the absence and in the presence of GAGs, such as metal ions, were discarded.

We chose the aggregation half-time (*t*_1/2_), that is the time at which the specific signal used to follow the aggregation reaction reaches half of its final value, to describe the kinetics of protein aggregation. *t*_1/2_ was preferred to the rate constant of elongation (*k*_agg_) or the lag phase duration (*t*_lag_) because the latter parameter cannot be compared in different experiments if the lag phase is absent. When only *k*_agg_ and *t*_lag_ were mentioned in the article, we used the following equation to calculate the *t*_1/2_ value [29]:
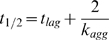
(1)For each set of experimental conditions, we calculated *G*, the natural logarithm of the ratio between the *t*_1/2_ values in the absence and in the presence of the GAG:
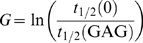
(2)Thus, if a GAG accelerates and decelerates the aggregation process *G* is positive and negative, respectively. In the absence of a lag phase, *G* is equal to Ln [*k*_agg_(GAG)/*k*_agg_(0)] (compare equations 1 and 2).

In cases where the authors of the original articles did not mention any kinetic parameters, but showed only kinetic traces, the in-house developed software plot2data was used to extract the data. The software allows the user to map a Cartesian 2-D space on a computer image containing a graph, in order to extracting the coordinates of interesting points and making them available as text values. The extracted data were then manually re-plotted, and the resulting plots were fitted to equations 3 or 4, depending on the absence or presence of a detectable lag phase, respectively [29]:
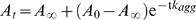
(3)

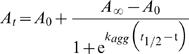
(4)where *A*_0_, *A_t_* and *A*_∞_ are the signal intensities of the techniques used to monitor aggregation at time 0, t, and ∞, respectively. *A*_0_, *A*_∞_, *k*_agg_ and *t*_1/2_ were used as floating parameters in the procedure of best fit.

The resulting dataset, summarizing the *G* values and the corresponding experimental conditions in which they where collected, is presented in Table S1 (see Supplementary Information).

### Multivariate analysis

For the multivariate analyses, *G* was set as the single dependent variable. Different parameters describing the GAGs, polypeptide chains and experimental characteristics were set as independent variables. These include, for the GAG, the number of sulfates per disaccharide unit, the number of negative charges per disaccharide unit, the chemical nature of the uronic acid (iduronic or glucuronic acid), the position of the sulfate (N- or O-sulfates), and the molecular weight; they also include, for the protein, the length, charge, composition in lysine and arginine residues, folding status (globular or natively unfolded proteins) and association with disease (disease-related or model proteins); finally they include the solute molarity and the protein∶GAG molar ratio for the experimental conditions. All the independent variables that were dichotomous (nature of the GAG uronic acid; position of the sulfates on the GAG; folding status of the protein; protein associated or not with disease) were recoded into dummy variables and their interaction terms with other variables were taken into account. We also systematically looked for the presence of possible quadratic effects for each continuous variable.

The multivariate analyses were performed with the Microsoft Excel add-on software PHStat2 [30], a tool that allows a statistically coherent construction and optimization of multivariate regression models. Both stepwise and best-subset model construction methods were used to reduce the number of significant variables. The final model was the one that best fulfilled the following characteristics: significance of each independent variable (*p*_variable_<0.05); significance of the model (*p*_model_<0.05); adjusted coefficient of determination (*R* ^2^_adj_) as close to 1 as possible; absence of collinearity between the different independent variables, detected with the variance inflation factor (VIF); homogeneous distribution of the residuals (homoscedasticity).

### Bootstrap and jackknife tests

Two statistical approaches were used to verify the significance and robustness of the chosen model. In the bootstrap test 100 subsets of the original dataset comprising 39 entries were randomly created, each time using 2/3 of the 39 entries (training sets, 26 entries each). Each of the 100 training sets was used to perform the same multivariate analysis previously performed on the whole dataset and to obtain a set of regression parameters. Each of the resulting 100 sets was then used in the predictive equation detailed below (equation 5, see results) to calculate *G* values on the remaining subset of 1/3 entries (test set, 13 entries). This led to the creation of 100 different sets of predicted and observed *G* values, that were evaluated by linear regression analysis to record correlation coefficients and p-values through goodness of fit F-statistic.

In the jackknife test, single entries were systematically removed from the full dataset of 39 entries and the multivariate analysis was repeated on shortened datasets of 38 entries (for a total of 39 steps), to obtain regression parameters with which we computed the predicted *G* value for the removed entry using equation 5 (see results). After the analysis was completed for the 39 removed entries the 39 predicted *G* values were plotted against the corresponding experimental values and the resulting plot was analyzed by means of a linear regression.

## Results

### General strategy

**Figure 1:**
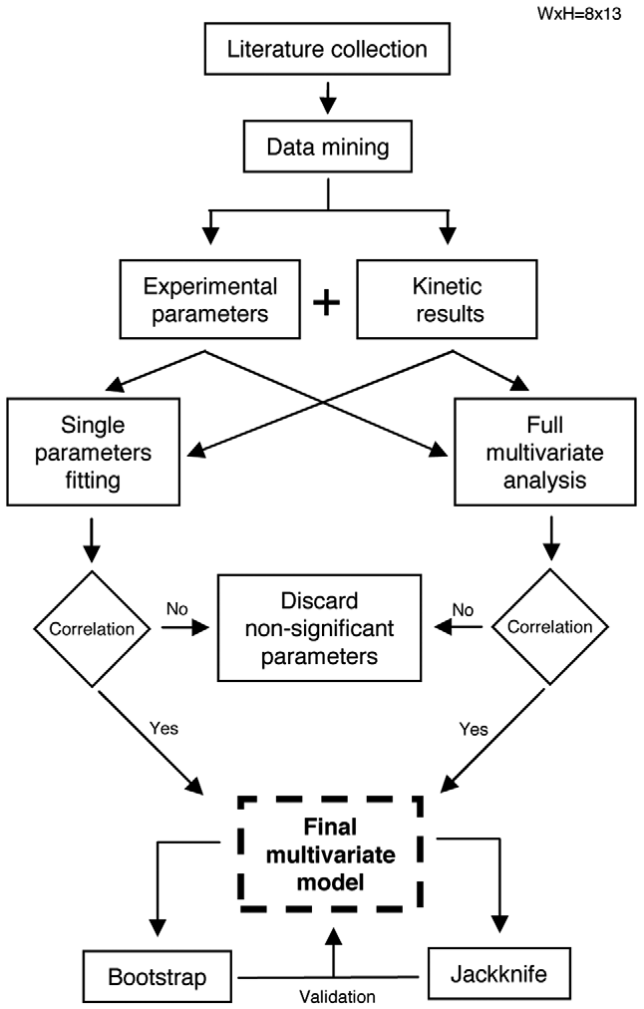
Scheme of the general strategy used in this study.

The general strategy adopted for this study is presented in Figure 1 (see also the *Methods* section). Briefly, experimental data reporting the effect of GAGs on the kinetics of amyloid fibril formation were collected from previously published articles using a precise and rigorous method, after an extensive search of the literature (Figure 1, step 1). The resulting dataset summarizes the effects of different GAGs on the aggregation kinetics of different proteins, together with the precise experimental conditions in which these effects were recorded in each case (GAG and protein types and concentrations; composition, ionic strength, total solute concentration, pH and temperature of the milieu). The effect of a GAG on protein aggregation was described by *G*, that is the natural logarithm of the ratio between the aggregation half-time *t*_1/2_ in the absence and in the presence of the GAG (see *Methods*). The resulting dataset comprises 39 sets of data, representing 8 different proteins, 16 different GAGs and a variety of experimental conditions (see Table S1 in Supplementary Information). The 8 proteins include both globular proteins, such as the immunoglobulin light chain variable domain, and natively unfolded proteins, such as a-synuclein. Some proteins are directly involved in disease, such as the β-amyloid peptide, while others are model proteins, like human muscle acylphosphatase. The 16 GAGs are either existing GAGs from different families, such as heparin or dermatan sulfate, or chemically modified GAGs such as fully desulfated heparin or dextran sulfate.

To identify the determinants responsible for the accelerating effects of GAGs on protein aggregation, we analyzed the influence of different parameters on *G*. This was done by performing in parallel single parameter fittings, through a search of correlations between *G* and a variety of parameters analyzed one by one (Figure 1, step 2a), and a multivariate analysis, that is a combination of different parameters as independent variables in a single equation to describe *G* as a function of all analysable parameters simultaneously (Figure 1, step 2b). The parameters that appeared from both step 2a and 2b to play a significant role on the GAG-mediated acceleration of protein aggregation were then combined into a single predictive equation yielding *G* as a function of the key parameters only (Figure 1, step 3). Finally, the validity and the robustness of the model and predictive equation were assessed by statistical tests (Figure 1, step 4).

### Single parameter analysis: characteristics of the GAGs

**Figure 2:**
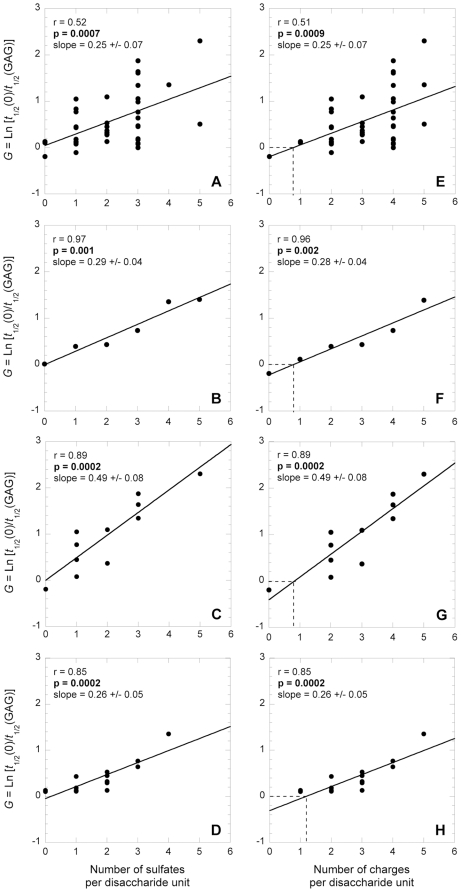
Influence of the number of sulfates and negative charges per GAG disaccharide unit on protein aggregation. A–D: dependence of the *G* value on the number of sulfates per GAG disaccharide unit; E–H: dependence of the *G* value on the number of negative charges per GAG disaccharide unit; A and E: different GAGs, proteins and experimental conditions; B and F: idem, but each *G* value in the plot is the mean of all the *G* values obtained with a GAG with the same number of sulfates or negative charges; C and G: only *G* data of α-synuclein in identical experimental conditions are plotted; D and H: only *G* data of the 173–243 fragment of gelsolin in identical experimental conditions are plotted. In all plots the solid lines represent the lines of best fit; the *r* and *p* values of the linear regression and the slope of the line of best fit are reported in each plot.

We first looked at the influence of the GAG sulfation state on *G*. When the *G* value was plotted against the number of sulfate moieties per GAG disaccharide unit for all the 39 entries of the dataset, a significant linear positive correlation was observed (Figure 2A, r = 0.52, *p* = 7.10^−4^). The analysis was repeated by plotting average *G* values, where each average *G* value is the mean of the *G* values related to the same sulfation state (Figure 2B). Again, the average *G* value was found to correlate significantly with the number of sulfates per disaccharide unit (Figure 2B, r = 0.97, *p* = 0.001). To limit the complications arising from the heterogeneity of proteins used in the study, we restricted the analysis to a single protein type, i.e. α-synuclein (Figure 2C) and the 173–243 fragment of gelsolin (Figure 2D), two polypeptides for which enough data were available for a statistical analysis. The correlation was found to be significant in both cases (Figure 2C,D, r = 0.89 and *p* = 2.10^−4^ in both cases). The high significance of the correlations shown in Figure 2A–D confirms the dependence of the *G* value on the sulfate state of the GAG and suggests that the sulfate moieties have comparable effects in the aggregation of the various proteins analyzed. Importantly, in all cases the straight line of best fit passes through the origin of the graph, where both the *x* and *y* variables have values of 0. This observation indicates that in the absence of sulfates the GAGs have no effects on the kinetics of protein aggregation. While these data demonstrate that the sulfation state of the GAG is a key determinant of the GAG-induced acceleration of protein aggregation, they also show that it is not the only one, as GAGs with the same number of sulfate groups per disaccharide units can have very different effects on protein aggregation (Figure 2A).

We then looked at the importance of the GAG negative charge in determining *G* (Figure 2E–H). The number of sulfates and the number of negative charges per disaccharide unit of a GAG are two highly correlated parameters, as each sulfate moiety brings 1 negative charge. However they are not identical, as most of the GAGs have one additional negative charge per disaccharide unit due to the presence of a carboxylate group. Significant correlations were observed between the *G* value and the number of charges per disaccharide unit whatever dataset was considered (Figure 2E–H). The slopes of the lines of best fit were found to be identical when *G* values are plotted versus the number of either sulfate moieties or negative charges (Figure 2A–H). However, in the latter plots the lines of best fit do not pass through the origins of the graphs, but have *G* values of 0 when the number of negative charge is *ca*. 1 (Figure 2E–H). This implies that the absence of effect on protein aggregation is observed when the GAGs carry one negative charge per disaccharide unit (i.e. only the carboxylate group) and no sulfates. Therefore, the correlation between the *G* value and the negative charge per disaccharide unit arises from the GAG sulfation state, with the carboxylate group appearing to have no effect.

**Figure 3:**
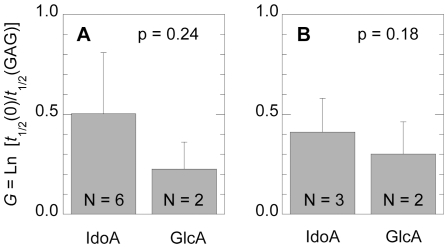
Influence of the chemical nature of the uronic acid present in the GAG on protein aggregation. A: different GAGs, proteins and experimental conditions; B: different GAGs, only the 173–243 fragment of gelsolin in identical experimental conditions. In both cases only GAGs with 2 sulfates per disaccharide unit are considered. Experimental errors indicate standard deviations. The high *p* values indicate lack of statistical significance.

The sulfate moieties in GAGs can be N- or O-sulfates. It has been proposed that N- and O-sulfates can have different effects on protein aggregation [31]. In our dataset, we did not observe any significant difference between the effects of N- or O-sulfated GAGs on protein aggregation kinetics (not shown). GAGs can also differ in terms of the type of the hexuronic acid, which can be either iduronic or glucuronic acid. It has been suggested that GAGs containing iduronic acid could be more active, due to the greater conformational flexibility of the iduronic pyranose ring with respect to the glucuronic pyranose ring [32]. However, we could not identify any significant difference between the effect of GAGs with iduronic or glucuronic acid on protein aggregation, when either all the data with the same GAG sulfation state were considered (Figure 3A) or when the analysis was restricted to data with the same sulfation state of GAG and only the 173–243 fragment of gelsolin as a polypeptide (Figure 3B). Finally, the *G* value was not found to correlate with the molecular weight of the GAG. Therefore, it seems that the sulfation state is the only GAG characteristic that has a significant effect on the GAG-mediated acceleration of amyloid fibril formation.

### Single parameter analysis: characteristics of the proteins

In a second step, we studied the influence of different parameters of the polypeptide chains. We looked at the effect of the protein length, charge, and composition in lysine and arginine residues, described in some cases to be responsible for GAG binding [6], [33]. We also divided the proteins of our dataset into globular or natively unfolded proteins, or into disease-related or disease-unrelated. We could not identify any significant correlation between *G* and any of these parameters, with any of the dataset used. This result could be due to the small number and heterogeneity of proteins in the database.

### Single parameter analysis: characteristics of the experimental conditions

**Figure 4:**
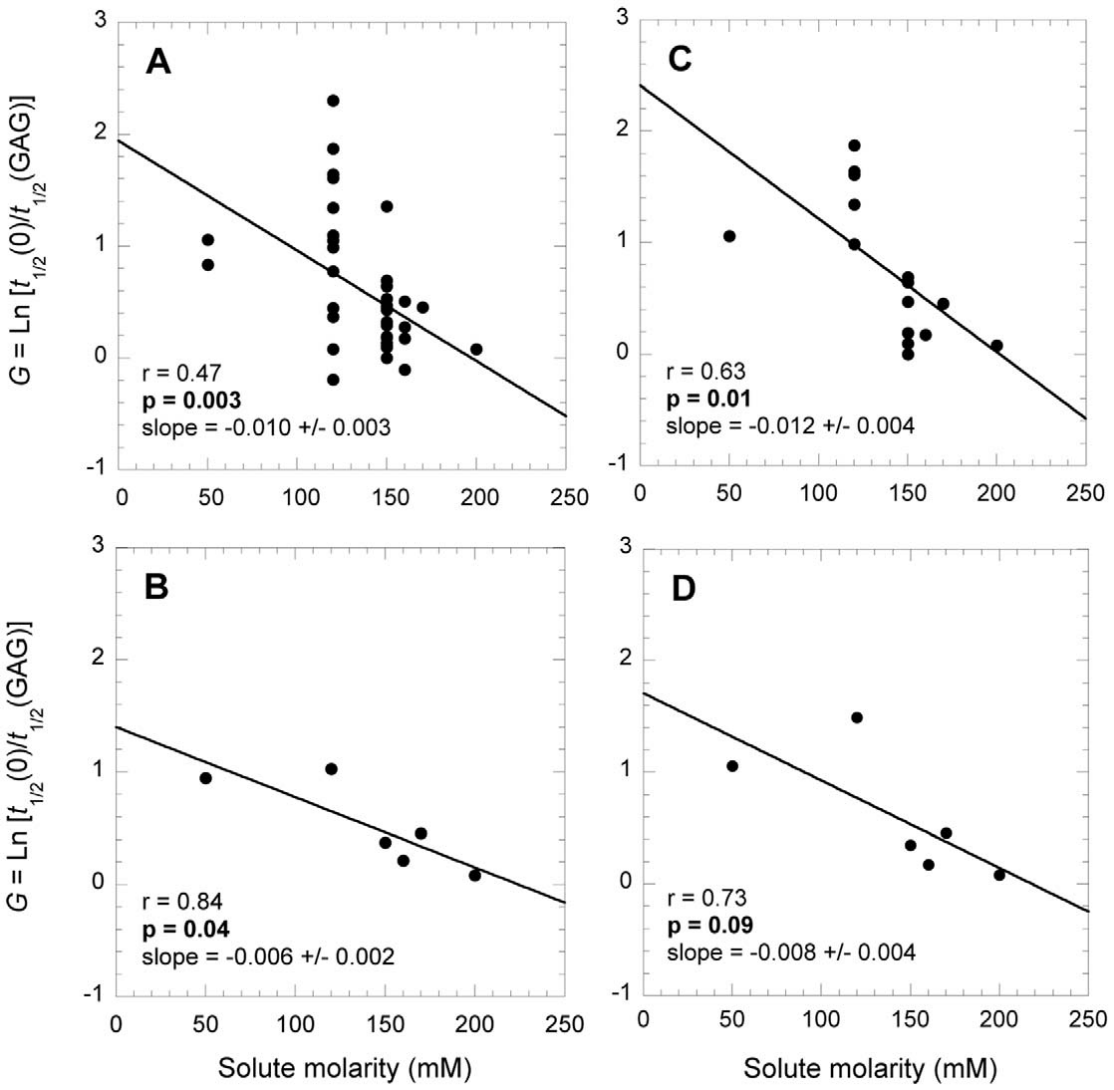
Influence of solute molarity on the GAG-mediated acceleration of amyloid fibril formation. A and B, different GAGs, proteins and experimental conditions; C and D, only heparin, different proteins, different experimental conditions; A and C, all *G* values; B and D, mean values of *G*, each calculated at a defined solute molarity range. In all plots the solid lines represent the lines of best fit; the *r* and *p* values of the linear regression and the slope of the line of best fit are reported in each plot.

We thoroughly analyzed the importance of the experimental conditions in determining the *G* value. Most of the experiments reported in our dataset were carried out at physiological temperature and pH, and under identical conditions of ionic strength (see Table S1). As a consequence, the influence of these three parameters could not be analyzed. To have an estimator of buffer composition that could be used as a descriptive parameter for our database, we analyzed the influence of the total solute concentration of the buffer. A significant negative correlation was found between the *G* value and the solute molarity when considering the entire dataset (Figure 4A, r = 0.47, *p* = 0.003). A higher solute molarity is associated with a less pronounced accelerating effect of the GAG on protein aggregation (Figure 4A). The analysis was repeated by plotting average *G* values, each calculated over a range of solute molarity, for the entire dataset; the analysis confirmed the presence of a correlation (Figure 4B, r = 0.84, *p* = 0.04). In order to limit the problems arising from the heterogeneity of the GAGs used, only data of the GAG heparin were considered in a subsequent analysis. A correlation was still observed when all *G* values obtained with heparin were plotted against solute molarity (Figure 4C, r = 0.63, *p* = 0.01), as well as when average *G* values, each calculated over a range of solute molarity, were plotted versus solute molarity (Figures 4D; r = 0.73, *p* = 0.09).

**Figure 5:**
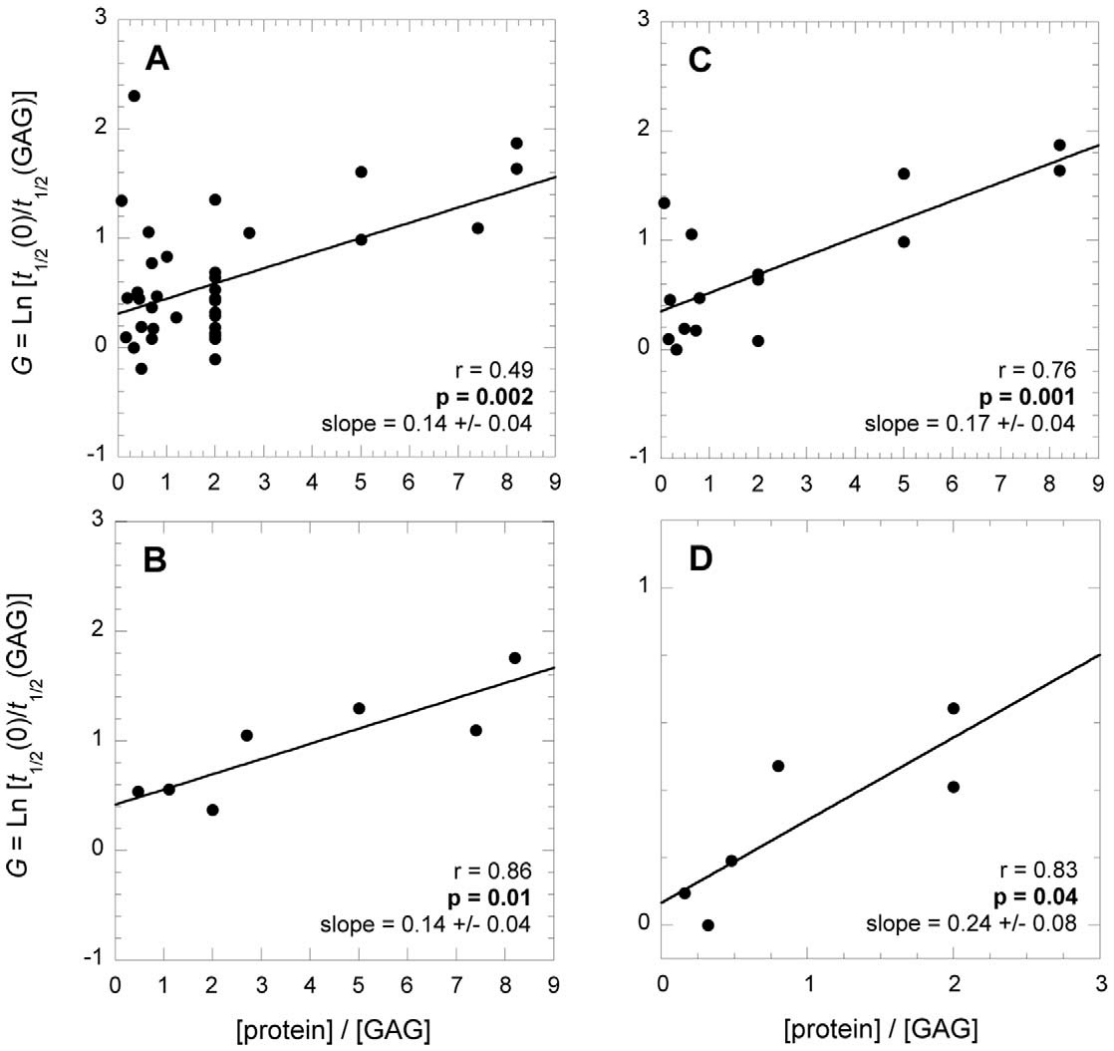
Influence of the protein∶GAG molar ratio on amyloid fibril formation. A, different GAGs, proteins and experimental conditions; B, idem but the mean values of *G*, each obtained at a given protein∶GAG molar ratio interval; C, only heparin, different proteins, different experimental conditions; D, only heparin, only the 173–243 fragment of gelsolin, different experimental conditions. In all plots the solid lines represent the lines of best fit; the *r* and *p* values of the linear regression and the slope of the line of best fit are reported in each plot.

The next studied parameter was the ratio of molar concentrations of the GAG and protein used in the experiments. A clear positive correlation existed between the *G* value and the protein∶GAG molar ratio using the whole dataset (Figure 5A, r = 0.49, *p* = 0.002), average *G* values calculated over intervals of protein∶GAG molar ratio (Figure 5B, r = 0.86, *p* = 0.01), only *G* values obtained with heparin (Figure 5C, r = 0.76, *p* = 0.001), or only *G* values obtained with heparin and the 173–243 fragment of gelsolin (Figure 5D, r = 0.83, *p* = 0.04). This finding shows that the GAG becomes more effective in accelerating amyloid formation if the concentration of protein grows more markedly than that of GAG. The possible origin of such a correlation will be discussed in the *Discussion* section.

### Multivariate analysis and construction of a predictive equation

We also performed a multivariate regression in parallel to, and independently of, the single parameter analyses. The parameters inserted in the multiparameter equations were the same as those analyzed individually. Thus, as far as the GAG is concerned, we considered the number of sulfate groups per disaccharide unit, the number of negative charges per disaccharide unit, the chemical nature of the uronic acid (iduronic or glucuronic acid), the type of the sulfate moiety (N- or O-sulfation) and the molecular weight for the GAG. As far as the protein is concerned, we took into account the protein length, net charge, composition in lysine and arginine residues, folding status (globular or natively unfolded proteins) and association with disease (disease-related or disease-unrelated). Finally, we considered for the experimental conditions the solute molarity and the protein∶GAG molar ratio. The multivariate regression was allowed to run on the entire dataset. The best model that fitted the experimental data was the following (see *Methods* for the definition of the best model):
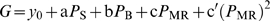
(5)where *P*_S_ is the number of sulfate groups per disaccharide unit, *P*_B_ is the total molarity of the solutes in mM units, *P*_MR_ is the protein∶GAG molar ratio and *y*_0_ is the *y* axis intercept. a, b, c and c′ are the multiplying factors of the various parameters and were left free to float in the fitting procedure, similarly to *y*_0_. The multivariate analysis yielded values of 2.0±0.7, 0.30±0.05, −0.016±0.004, 0.11±0.03 and −0.0020±0.0005, for *y*_0_, a, b, c and c′, respectively. Note that this model includes a quadratic effect of the protein∶GAG molar ratio. Models that did not consider this quadratic effect, i.e. where the c′(*P*_MR_)^2^ term was absent, were much less accurate in fitting to the experimental data.

The model resulting from the multivariate analysis is highly significant. All the coefficients of the single variables have a significance lower than 10^−3^; the significance of the whole model is equal to 2.10^−6^; the adjusted *R* ^2^ value of the model is equal to 0.74, indicating that 74% of the variance observed in the experimental dataset is explained by this simple model. Finally, we performed bootstrap and jackknife tests that verified the robustness of the model and its independence of the dataset composition (see Figures S1 and S2 in Supplementary Information).

The results of the multivariate analysis have two main implications. First, it confirms the significance of the three parameters identified with the single parameter analyses in determining the GAG-mediated acceleration of amyloid fibril formation: the sulfation state of the GAG, the molarity of the solutes, and the protein∶GAG molar ratio. Second, it confirms that all the other parameters studied in the single parameter analyses do not have a similar importance and appear to be non-significant altogether (probably as a result of the small number of entries in our dataset, at least in some cases).

**Figure 6:**
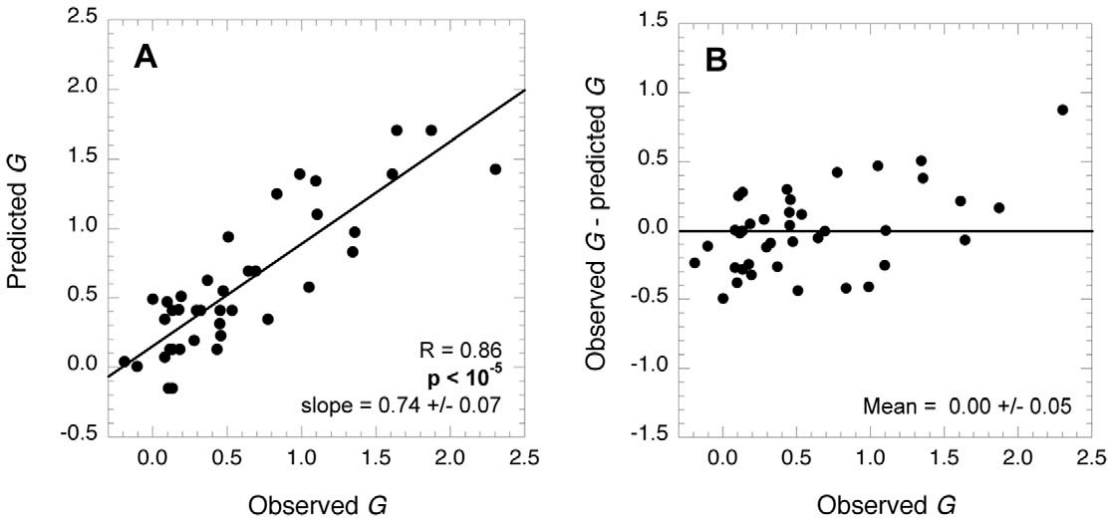
Prediction of the effect of GAGs on amyloid fibril formation using the predictive **equation 5**. A, Predicted values of *G* plotted *versus* those observed experimentally. The solid line represents the straight line of best fit; the *r* and *p* values of the linear regression and the slope of the line of best fit are reported in the plot. B, Residuals between *G* values observed experimentally and predicted plotted *versus* the *G* values observed experimentally. The solid line represents the mean of the residuals.

Finally, we used equation 5 to predict *G* based solely on the knowledge of the sulfation state of the GAG, the molarity of the solutes, and the protein∶GAG molar ratio, for the 39 entries of the dataset (Figure 6). The values of *G* predicted by equation 5 correlate significantly with those measured experimentally, as shown in Figure 6A (*r* = 0.86; *p*<10^−5^). The residuals between the *G* values observed experimentally and those predicted by equation 5 are small and randomly distributed around 0, confirming the validity of the model (Figure 6B).

## Discussion

In this work we have used previously published data to build a large database containing the effects of various GAGs on the rate of amyloid fibril formation by different proteins and in different solution conditions. The aim was to identify and rationalize the chemical factors involved in the GAG-induced acceleration of the process of amyloid fibril formation. We have adopted two different and complementary methods for identifying such factors: a set of single parameter analyses and a multivariate analysis. Using this approach, we have identified three major determinants of the effect of GAGs on the kinetics of amyloid formation: the sulfation state of the GAG, the molar concentration of all compounds present in the buffer, and the protein/GAG molar ratio. It is highly significant that the two strategies have identified the same parameters, reinforcing the conclusions. The results do not rule out the importance of additional factors, particularly those arising from the chemical nature and structure of the protein undergoing aggregation. However, our statistical approach could not identify any of such determinants, most probably because of the limited size of our database.

### The importance of the GAG sulfation state

It has been previously pointed out that the sulfation state of a GAG is an important determinant of the ability of the polysaccharide to promote or accelerate amyloid fibril formation [5], [6], [18], [22], [23], [34], [35]. A correlation between the sulfation state of the GAG and the extent of the amyloid formation acceleration has been observed using various systems, including the islet amyloid polypeptide [18], the amyloid β peptide [35], β2-microglobulin [36], the 173–243 fragment of gelsolin [5], α-synuclein [6] and an immunoglobulin light-chain variable domain [23]. However, in all these cases it has not been possible to distinguish between the sulfation state and the charge state (also involving the carboxylate group of the GAG), leading some investigators to emphasize, more generally, the importance of the charge state of the GAG rather than of the sulfation state [18], [36]. Moreover, in previous studies it has not been possible to clarify whether the backbone of the polysaccharide plays a role in the GAG-protein interaction. Our observation that the GAG-induced acceleration disappears when the sulfation state is zero indicates that neither the carboxylate moiety, nor the backbone of the polysaccharide play relevant roles in the effect of GAGs on amyloid formation.

In addition, our comparison between GAGs containing iduronic and glucuronic acids has showed no significant differences, indicating that the configuration of the chiral carbon 5 bearing the carboxylate group in the uronic acid residue has no apparent importance in determining the effect of the GAG. Similarly, no differences have been observed when comparing O- and N-sulfates. The finding that sulfate moieties play a role due to their high density and their regular distribution on the polysaccharide surface [37] indicates that the distinction between N- and O-sulfation might not be a fundamental one.

Finally, we have not observed any effect of the GAG molecular weight. It should be noticed that we have considered only polysaccharides with a sufficiently high length. Oligosaccharides shorter than 6 or 8 disaccharide units have been shown to have a lower effect on protein aggregation than longer GAGs [29], [37], [38]. Thus, it appears that the GAG loses its effect only below a well defined threshold, when the excessively small length of the polysaccharide chain suppresses the macromolecular nature of the GAG.

### The ratio of protein to GAG concentration as a critical factor

One of the clearest result of our analysis is a strong dependence of the accelerating effect of the GAG on the respective protein and GAG molar concentrations. This parameter is ignored in all studies aimed at investigating the effect of GAGs on protein aggregation and could explain some discrepancies observed between different sets of experiments, for example those involving α-synuclein [6], [39].

The correlation observed in the single parameter analysis implies that an excess of GAG decreases its accelerating effect on protein aggregation. The multivariate analysis indicates the existence of a negative quadratic component, in addition to a positive linear component, in the dependence of the acceleration of protein aggregation on the protein∶GAG molar ratio. This result translates into a bell-shape dependence of the acceleration on the protein∶GAG molar ratio. In such a dependence the effect of GAG on protein aggregation is maximal at a given protein∶GAG molar ratio. At lower or higher values of the protein∶GAG molar ratio the GAG has a lower effect on protein aggregation. From the collected experimental data and the resulting multivariate analysis we can estimate this ratio as 10, that is 1 GAG molecule per 10 protein molecules. The descending arm of the dependence – a decreased effect when the GAG concentration increases – could originate from the ability of the GAG molecules to sequester protein molecules at different and distant sites, hindering their effective interaction and aggregation. Importantly, most of the experimental data reported so far in the literature and collected here were performed in the descending arm, i.e. at high GAG concentrations (see Figure 5 and Table S1 in Supplementary Information).

At the high concentration of GAGs and at the relatively low concentration of soluble, non-fibrous proteins populating the basement membrane of the extracellular matrix, where amyloid fibril formation occurs in pathology, GAGs may have an effect much lower than previously thought, without producing a remarkable acceleration of amyloid fibril formation. In such conditions of low protein∶GAG molar ratio, protein aggregation still occurs in proximity of the GAGs, given the high affinity of such compounds for proteins, but the polysaccharides may have a neutral effect, rather than an accelerating potential. On the other hand, a high local concentration of peptide/protein may occur at the sites at which it is secreted. This issue deserves further analysis.

### The importance of the solute molarity

Another result emerging from our analysis is that the ability of GAGs to accelerate amyloid fibril formation correlates negatively with the molarity of the compounds composing the buffer solution. Such a negative correlation, which has already been reported on isolated systems [20], [40]–[42], is shown here to be a generic phenomenon of the protein-GAG interaction. The dependence of the GAG-mediated acceleration of protein aggregation on the solute molarity can originate from two non-exclusive phenomena. It first reveals that the interactions between GAGs and proteins are in part electrostatic, as these interactions are shielded by high salt concentrations. It could also be due to the release of the GAG positive counterion upon protein binding, with such a release being entropically favored by a low ionic strength buffer [43], [44].

Intriguingly, at the salt concentrations existing in the human extracellular fluids amyloid fibril formation seems to be unaffected or only weakly affected by GAGs (see Figure 4). This observation reinforces the aforementioned possibility that under the conditions found in the basement membrane of the extracellular matrix GAGs may not have that dramatic accelerating effect on protein aggregation.

### Conclusions

The three parameters identified here using both single parameter and multivariate analyses have been combined into a single predictive equation of the effect of GAGs on the kinetics of amyloid formation. The equation accounts for ¾ of the observed experimental variability in the observed acceleration, with the remaining ¼ arising from other characteristics that are yet unidentified. Such unidentified factors could be inherent structural and/or sequence-based characteristics of the protein, as well as other determinants of the environment or of the GAG structure. The further improvement of our mathematical tool awaits accumulation of experimental data on larger sets of proteins, GAGs and conditions.

It is still remarkable, however, to have achieved a predictive mathematical tool that can determine, with reasonable accuracy, the effect of a given GAG on the amyloid fibril formation process of a given protein and under well-defined experimental conditions. Albeit important, the outcome of the analysis is not limited to the obtainment of a predictive algorithm. It has identified previously neglected factors as important determinants of the GAG-mediated acceleration of protein aggregation, such as solute molarity and protein∶GAG molar ratio. The analysis has highlighted that a GAG is not necessarily pro-aggregating, but can rather have different effects depending on the conditions, and has showed that under the conditions existing in the basement membrane of the extracellular matrix, where amyloid structures deposit in pathology, GAGs can have little effect on the process of amyloid fibril formation.

## Supporting Information

Figure S1 Results from the bootstrap test. The dataset was randomly subsampled generating 100 training sets (containing 2/3 of the data corresponding to 26 entries) and 100 test sets (containing the remaining 1/3 of the data corresponding to 13 entries). Each training set was subjected to multivariate analysis as described for the full dataset (see Methods) to generate a predictive equation with its own set of parameters, that was then applied to the corresponding test set to obtain G values predictions. The 100 bootstrap tests performed are represented on the x axis. The closed circles indicate the p values of the 100 model predictive equations built from the training sets (the scale is reported on the left y axis). The open circles indicate the p-values of the regressions obtained plotting predicted versus observed G values for the 100 test sets. The mean and associated standard error values of the Pearson coefficients associated to the p-regression values are R = 0.789±0.008, indicating that the model we built was robust in term of dataset composition.
(0.54 MB TIF)

Figure S2 Results from the jackknife test. For each of the 39 data of our dataset, the predicted G value was calculated applying the predictive equation generated by the multivariate regression analysis on a dataset composed of the 38 remaining data (see Methods). The graph shows the linear correlation analysis between the 39 predicted vs experimental G values, giving a significant correlation with an R^2^ = 0.59 (p-value<10^−5^).
(0.36 MB TIF)

Table S1 Database of the effects of GAGs on the kinetics of protein aggregation, constructed from the literature. ^a^Dummy variables. The binary code indicated is the one used for the multivariate analyses. ^b^Protein net charge calculated at pH7.5. References: Calamai et al (2006) Biochemistry 45:12806 - Cohlberg et al (2002) Biochemistry 41:1502 - McLaughlin et al (2006) Protein Sci 15:1710 - McLaurin et al (1999) Eur J Biochem 266:1101 - Shuvaev and Siest (2000) Neurosci Lett 280:131 - Suk et al (2006) Biochemistry 45:2234 - Takase (1998) FEBS Lett 441:271 - Uversky et al (2005) Brain Res Mol Brain Res 134:84.
(0.06 MB PDF)
