# Supplementary figures and images for: A Computational Approach for Identifying the Chemical Factors Involved in the Glycosaminoglycans-Mediated Acceleration of Amyloid Fibril Formation
Source: PLoS One. 2010 Jun 29;5(6):e11363. doi: 10.1371/journal.pone.0011363 (PMC2894048; doi:10.1371/journal.pone.0011363)

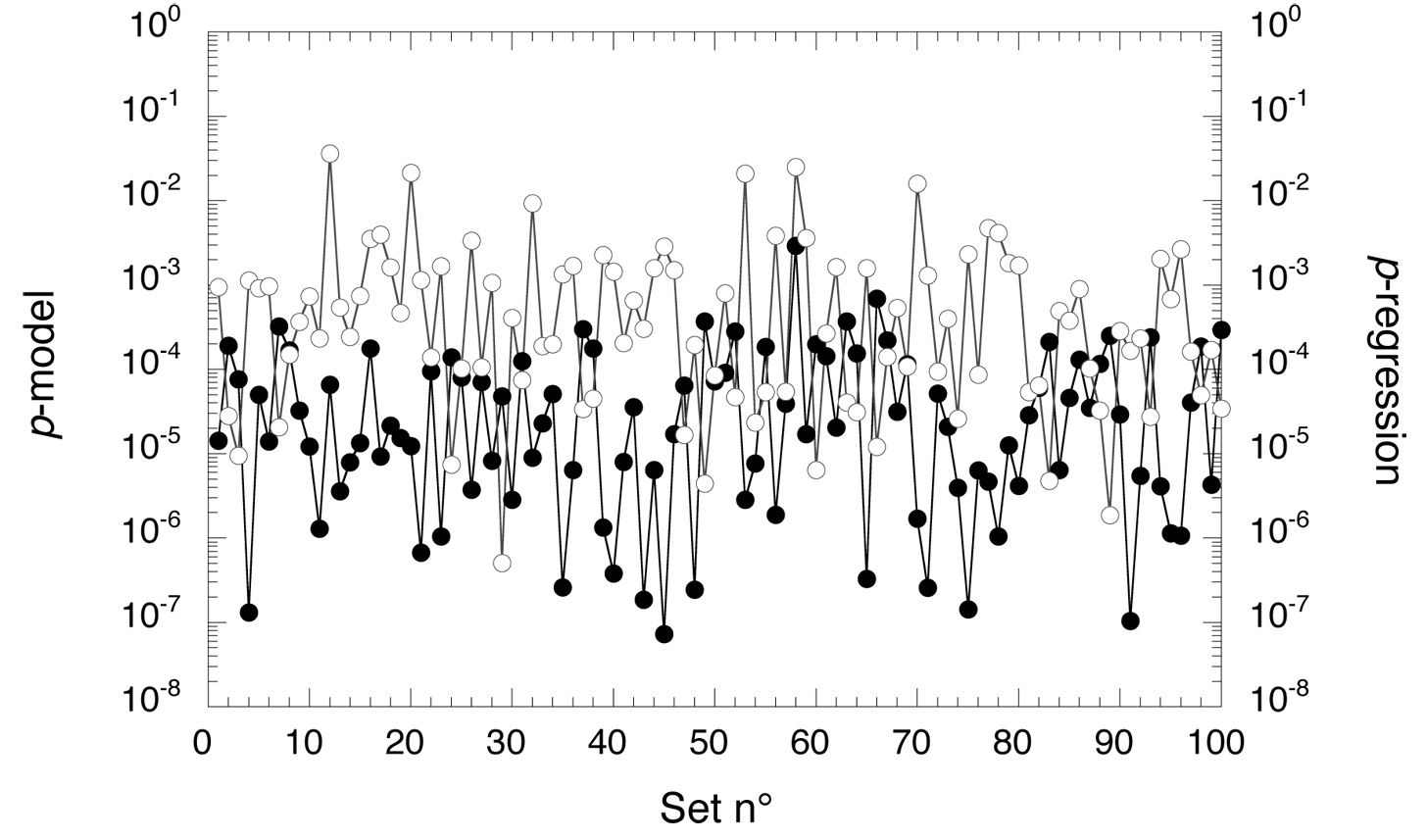

Supplement: Figure S1 — Results from the bootstrap test. The dataset was randomly subsampled generating 100 training sets (containing 2/3 of the data corresponding to 26 entries) and 100 test sets (containing the remaining 1/3 of the data corresponding to 13 entries). Each training set was subjected to multivariate analysis as described for the full dataset (see Methods) to generate a predictive equation with its own set of parameters, that was then applied to the corresponding test set to obtain G values predictions. The 100 bootstrap tests performed are represented on the x axis. The closed circles indicate the p values of the 100 model predictive equations built from the training sets (the scale is reported on the left y axis). The open circles indicate the p-values of the regressions obtained plotting predicted versus observed G values for the 100 test sets. The mean and associated standard error values of the Pearson coefficients associated to the p-regression values are R = 0.789±0.008, indicating that the model we built was robust in term of dataset composition. (0.54 MB TIF) [file pone.0011363.s001.tif]

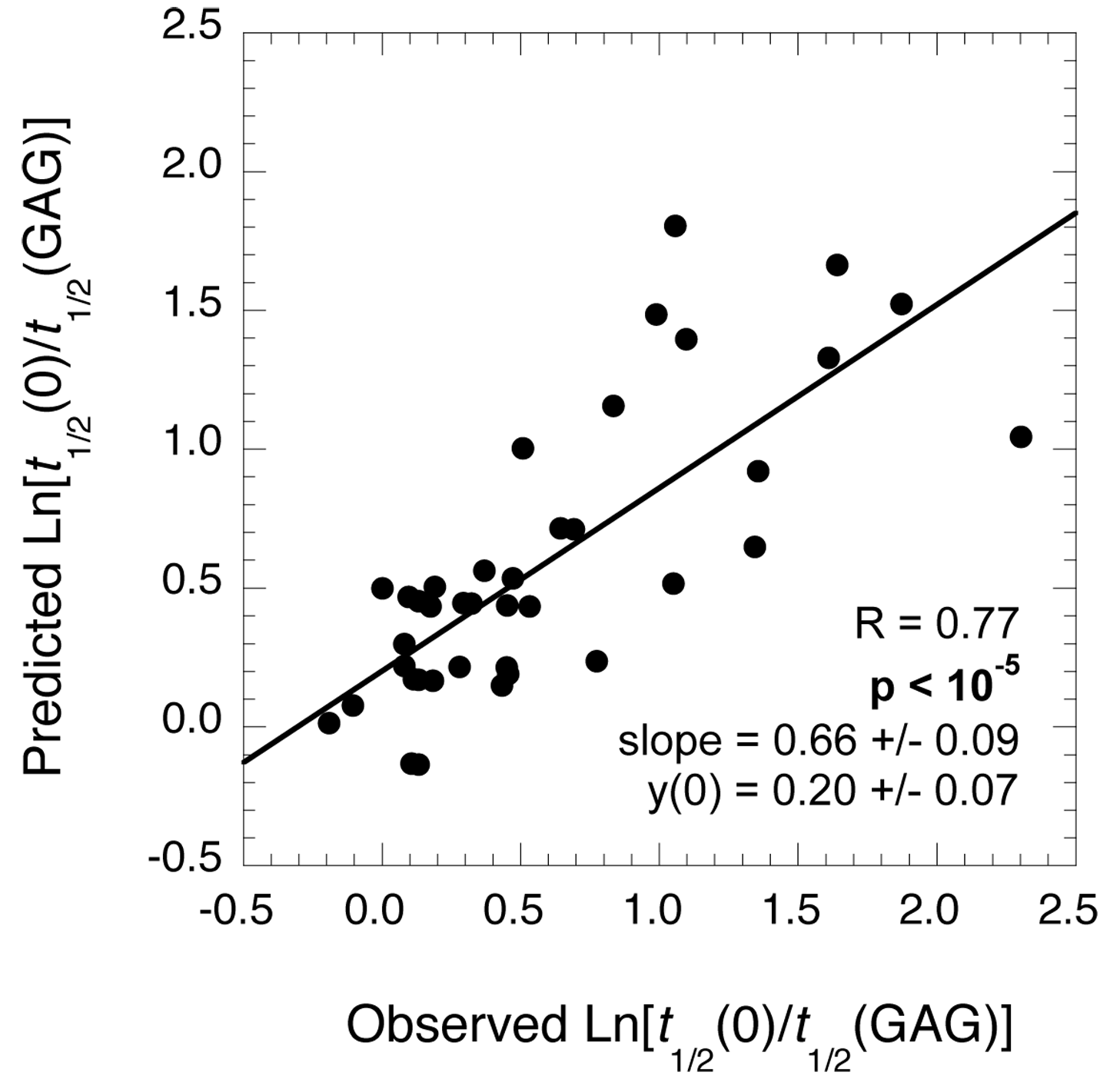

Supplement: Figure S2 — Results from the jackknife test. For each of the 39 data of our dataset, the predicted G value was calculated applying the predictive equation generated by the multivariate regression analysis on a dataset composed of the 38 remaining data (see Methods). The graph shows the linear correlation analysis between the 39 predicted vs experimental G values, giving a significant correlation with an R2 = 0.59 (p-value<10−5). (0.36 MB TIF) [file pone.0011363.s002.tif]
