# Supplementary material for: A Computational Approach for Identifying the Chemical Factors Involved in the Glycosaminoglycans-Mediated Acceleration of Amyloid Fibril Formation
Source: PLoS One. 2010 Jun 29;5(6):e11363. doi: 10.1371/journal.pone.0011363 (PMC2894048; doi:10.1371/journal.pone.0011363)
Supplement: Table S1 — Database of the effects of GAGs on the kinetics of protein aggregation, constructed from the literature. aDummy variables. The binary code indicated is the one used for the multivariate analyses. bProtein net charge calculated at pH7.5. References: Calamai et al (2006) Biochemistry 45:12806 - Cohlberg et al (2002) Biochemistry 41:1502 - McLaughlin et al (2006) Protein Sci 15:1710 - McLaurin et al (1999) Eur J Biochem 266:1101 - Shuvaev and Siest (2000) Neurosci Lett 280:131 - Suk et al (2006) Biochemistry 45:2234 - Takase (1998) FEBS Lett 441:271 - Uversky et al (2005) Brain Res Mol Brain Res 134:84. (0.06 MB PDF) [file pone.0011363.s003.pdf]

Reference	G = Ln $[t_{1/2}(0)/t_{1/2}(\mathrm{GAG})]$	GAG Name	N SO3- /diS	N charge /diS	IdoA (1) vs. GlcA (0) [a]	N- (1) vs. O-(0) sulfatation [a]	MW (kD)	PROTEIN Name	Length (aa)	Charge [b]	% Lys+ Arg	Folded (1) vs. NUP (0) [a]	Disease (1) vs. no (0) [a]	EXPERIMENTAL CONDITIONS pH	T (°C)	Buffer composition	Buffer ionic strength (mM)	Solute molarity (mM)	protein :GAG molar ratio
Calamai et al, 2006	2.713	heparin	3	-4	1	1	18	mAcP	98	+5.7	15.3	1	0	5.5	25	50 mM sodium acetate	44	50	0.62
McLaurin et al, 1999	0.833	chondroitin-4-sulfate	1	-2	0	0	n.d.	Aβ40	40	-2.4	7.5	0	1	7.0	25	50 mM sodium phosphate	108	50	1.00
Cohlberg et al, 2002	1.639	heparin	3	-4	1	1	18	α-synuclein (wt)	140	-9.2	10.7	0	1	7.5	37	20 mM Tris, 100 mM NaCl	113	120	8.24
Cohlberg et al, 2002	1.872	heparin	3	-4	1	1	18	α-synuclein (wt)	140	-9.2	10.7	0	1	7.5	37	20 mM Tris, 100 mM NaCl	113	120	8.24
Cohlberg et al, 2002	1.05	heparan sulfate	1	-2	1	0	7,5	α-synuclein (wt)	140	-9.2	10.7	0	1	7.5	37	20 mM Tris, 100 mM NaCl	113	120	2.66
Cohlberg et al, 2002	1.095	N-acetylated heparin	2	-3	1	0	18	α-synuclein (wt)	140	-9.2	10.7	0	1	7.5	37	20 mM Tris, 100 mM NaCl	113	120	7.36
Cohlberg et al, 2002	0.368	dermatan sulfate	2	-3	1	0	n.d.	α-synuclein (wt)	140	-9.2	10.7	0	1	7.5	37	20 mM Tris, 100 mM NaCl	113	120	0.69
Cohlberg et al, 2002	0.08	chondroitin-4-sulfate	1	-2	0	0	n.d.	α-synuclein (wt)	140	-9.2	10.7	0	1	7.5	37	20 mM Tris, 100 mM NaCl	113	120	0.69
Cohlberg et al, 2002	0.773	chondroitin-6-sulfate	1	-2	0	0	n.d.	α-synuclein (wt)	140	-9.2	10.7	0	1	7.5	37	20 mM Tris, 100 mM NaCl	113	120	0.69
Cohlberg et al, 2002	0.449	keratan sulfate	1	-2	0	0	n.d.	α-synuclein (wt)	140	-9.2	10.7	0	1	7.5	37	20 mM Tris, 100 mM NaCl	113	120	0.43
Cohlberg et al, 2002	2.303	dextran sulfate	5	-5	0	0	10	α-synuclein (wt)	140	-9.2	10.7	0	1	7.5	37	20 mM Tris, 100 mM NaCl	113	120	0.33
Cohlberg et al, 2002	-0.192	dextran	0	0	0	0	10	α-synuclein (wt)	140	-9.2	10.7	0	1	7.5	37	20 mM Tris, 100 mM NaCl	113	120	0.48
Cohlberg et al, 2002	1.609	heparin	3	-4	1	1	18	α-synuclein (A30P)	140	-9.2	10.7	0	1	7.5	37	20 mM Tris, 100 mM NaCl	113	120	4.99
Cohlberg et al, 2002	0.987	heparin	3	-4	1	1	18	α-synuclein (A53T)	140	-9.2	10.7	0	1	7.5	37	20 mM Tris, 100 mM NaCl	113	120	4.99
Suk et al, 2006	0.69	heparin	3	-4	1	1	5	gelsolin	71	+0.3	11.3	0	1	7.0	37	50 mM HEPES, 100 mM NaCl	115	150	2.00
Suk et al, 2006	0.432	heparan sulfate	1	-2	1	0	n.d.	gelsolin	71	+0.3	11.3	0	1	7,0	37	50 mM HEPES, 100 mM NaCl	115	150	2.00
Suk et al, 2006	0.451	dermatan sulfate	2	-3	1	0	n.d.	gelsolin	71	+0.3	11.3	0	1	7.0	37	50 mM HEPES, 100 mM NaCl	115	150	2.00
Suk et al, 2006	0.182	chondroitin-4-sulfate	1	-2	0	0	n.d.	gelsolin	71	+0.3	11.3	0	1	7.0	37	50 mM HEPES, 100 mM NaCl	115	150	2.00
Suk et al, 2006	0.113	chondroitin-6-sulfate	1	-2	0	0	n.d.	gelsolin	71	+0.3	11.3	0	1	7.0	37	50 mM HEPES, 100 mM NaCl	115	150	2.00
Suk et al, 2006	0.131	chondroitin-2,6-sulfate	2	-3	0	0	n.d.	gelsolin	71	+0.3	11.3	0	1	7.0	37	50 mM HEPES, 100 mM NaCl	115	150	2.00
Suk et al, 2006	0.322	chondroitin-4,6-sulfate	2	-3	0	0	n.d.	gelsolin	71	+0.3	11.3	0	1	7.0	37	50 mM HEPES, 100 mM NaCl	115	150	2.00
Suk et al, 2006	0.131	keratan sulfate	1	-2	0	0	n.d.	gelsolin	71	+0.3	11.3	0	1	7.0	37	50 mM HEPES, 100 mM NaCl	115	150	2.00
Suk et al, 2006	0.131	hyaluronic acid	0	-1	0	0	n.d.	gelsolin	71	+0.3	11.3	0	1	7.0	37	50 mM HEPES, 100 mM NaCl	115	150	2.00
Suk et al, 2006	0.104	fully desulfated heparin	0	-1	1	0	5	gelsolin	71	+0.3	11.3	0	1	7.0	37	50 mM HEPES, 100 mM NaCl	115	150	2.00
Suk et al, 2006	0.293	N-desulfated heparin	2	-3	1	0	5	gelsolin	71	+0.3	11.3	0	1	7.0	37	50 mM HEPES, 100 mM NaCl	115	150	2.00
Suk et al, 2006	0.531	2O-desulfated heparin	2	-3	1	1	5	gelsolin	71	+0.3	11.3	0	1	7.0	37	50 mM HEPES, 100 mM NaCl	115	150	2.00
Suk et al, 2006	1.356	fully-O-desulfated heparin	4	-5	1	1	5	gelsolin	71	+0.3	11.3	0	1	7.0	37	50 mM HEPES, 100 mM NaCl	115	150	2.00
Suk et al, 2006	0.642	heparin	3	-4	1	1	15	gelsolin	71	+0.3	11.3	0	1	7.0	37	50 mM HEPES, 100 mM NaCl	115	150	2.00
Suk et al, 2006	0.095	heparin	3	-4	1	1	1.186	gelsolin	71	+0.3	11.3	0	1	7.0	37	50 mM HEPES, 100 mM NaCl	115	150	0.16
Suk et al, 2006	0,000	heparin	3	-4	1	1	2.372	gelsolin	71	+0.3	11.3	0	1	7.0	37	50 mM HEPES, 100 mM NaCl	115	150	0.32
Suk et al, 2006	0.191	heparin	3	-4	1	1	3.558	gelsolin	71	+0.3	11.3	0	1	7.0	37	50 mM HEPES, 100 mM NaCl	115	150	0.48
Suk et al, 2006	0.472	heparin	3	-4	1	1	5.93	gelsolin	71	+0.3	11.3	0	1	7.0	37	50 mM HEPES, 100 mM NaCl	115	150	0.80
Uversky et al, 2005	1.343	heparin	3	-4	1	1	n.d.	α-synuclein (wt)	140	-9.2	10.7	0	1	7.5	37	20 mM sodium phosphate, 100 mM NaCl	152	120	0.07
McLaughlin et al, 2006	0.174	heparin	3	-4	1	1	18	V_L AL-09	108	-3.1	6.5	1	1	7.4	37	10 mM Tris, 150 mM NaCl	157	160	0.72
McLaughlin et al, 2006	-0.105	chondroitin-4-sulfate	1	-2	0	0	50	V_L AL-09	108	-3.1	6.5	1	1	7.4	37	10 mM Tris, 150 mM NaCl	157	160	2.00
McLaughlin et al, 2006	0.278	dermatan sulfate	2	-3	1	0	30	V_L AL-09	108	-3.1	6.5	1	1	7.4	37	10 mM Tris, 150 mM NaCl	157	160	1.20
McLaughlin et al, 2006	0.507	dextran sulfate	5	-5	0	0	10	V_L AL-09	108	-3.1	6.5	1	1	7.4	37	10 mM Tris, 150 mM NaCl	157	160	0.40
Takase,1998	0.455	heparin	3	-4	1	1	3	HEL	129	+7.1	13.2	1	0	7.5	25	20 mM Tris, 150 mM NaCl	166	170	0.19
Shuvaev and Siest, 2000	0.080	heparin	3	-4	1	1	6	Aβ40	40	-2.9	7.5	0	1	7.4	25	50 mM Tris, 150 mM Nacl	192	200	2.00

Table S1. Database of the effects of GAGs on the kinetics of protein aggregation, constructed from the literature.

[a] Dummy variables. The binary code indicated is the one used for the multivariate analyses.

[b] protein net charge calculated at pH7.5

References:

Calamai M, Kumita JR, Mifsud J, Parrini C, Ramazzotti M, Ramponi G, Taddei N, Chiti F, Dobson CM (2006) Nature and significance of the interactions between amyloid fibrils and biological polyelectrolytes. Biochemistry 45: 12806-12815.

Cohlberg JA, Li J, Uversky VN, Fink AL (2002) Heparin and other glycosaminoglycans stimulate the formation of amyloid fibrils from alpha-synuclein in vitro. Biochemistry 41: 1502-1511.

McLaughlin RW, De Stigter JK, Sikkink LA, Baden EM, Ramirez-Alvarado M (2006) The effects of sodium sulfate, glycosaminoglycans, and Congo red on the structure, stability, and amyloid formation of an immunoglobulin light-chain protein. Protein Sci 15: 1710-1722.

McLaurin J, Franklin T, Zhang X, Deng J, Fraser PE (1999) Interactions of Alzheimer amyloid-beta peptides with glycosaminoglycans effects on fibril nucleation and growth. Eur J Biochem 266: 1101-1110.

Shuvaev VV, Siest G (2000) Heparin specifically inhibits binding of apolipoprotein E to amyloid beta-peptide. Neurosci Lett 280: 131-134.

Suk JY, Zhang F, Balch WE, Linhardt RJ, Kelly JW (2006) Heparin accelerates gelsolin amyloidogenesis. Biochemistry 45: 2234-2242.

Takase K (1998) Reactions of denatured proteins with other cellular components to form insoluble aggregates and protection by lactoferrin. FEBS Lett 441: 271-274.

Uversky VN, Yamin G, Munishkina LA, Karymov MA, Millett IS, Doniach S, Lyubchenko YL, Fink AL (2005) Effects of nitration on the structure and aggregation of alpha-synuclein. Brain Res Mol Brain Res 134: 84-102.
